# Knowledge about and sources of smoking-related knowledge, and influencing factors among male urban secondary school students in Chongqing, China

**DOI:** 10.1186/s40064-016-3589-z

**Published:** 2016-10-26

**Authors:** Xianglong Xu, Cheng Chen, Abu S. Abdullah, Manoj Sharma, Hengyi Liu, Yong Zhao

**Affiliations:** 1 School of Public Health and Management, Chongqing Medical University, Chongqing, 400016 China; 2 Research Center for Medicine and Social Development, Chongqing Medical University, Chongqing, 400016 China; 3 Innovation Center for Social Risk Governance in Health, Chongqing Medical University, Chongqing, 400016 China; 4 Xiangya School of Public Health, Central South University, Changsha, 410001 Hunan Province China; 5 Global Health Program, Duke Kunshan University, Kunshan, 215347 Jiangsu Province China; 6 Department of General Internal Medicine, School of Medicine, Boston University Medical Center, Boston, MA 02118 USA; 7 Behavioral and Environmental Health, Jackson State University, Jackson, MS USA

**Keywords:** Smoking-related knowledge, Sources, Influencing factors, Hobby, Male, Secondary school students, China

## Abstract

**Objectives:**

This study examined smoking-related knowledge, sources of smoking-related knowledge and its influencing factor among male urban secondary school students.

**Methods:**

We conducted a cross-sectional survey, using a self-administered questionnaire, among 1297 male secondary school students in municipal areas of Chongqing, China.

**Results:**

Non-smokers had a better knowledge of smoking hazards than smokers. Less than 20% of students knew that smoking can cause heart disease, peptic ulcer, and cerebral stroke. Sources of smoking-related knowledge differed between smokers and non-smokers, respectively: TV (76.5 vs. 76.7%), teacher (70.1 vs. 62.4%), social network (66.6 vs. 73.1%), parents (57.0 vs. 59.2%), newspaper (55.4 vs. 61.6%), and friends (37.7 vs. 33.9%). Non-smokers’ hobbies of reading were significantly higher than smokers’ (χ^2^ = 11.5845, p = 0.0007). Smokers’ hobbies of online games (χ^2^ = 14.9106, p = 0.0001), and sports (χ^2^ = 4.8609, p = 0.0275) were significantly higher than non-smokers’. Generalized estimating equations (GEE) found that students whose both father and mother smoke were unable to receive tobacco-related knowledge. GEE also found that students in high school, with affluent family economic condition and with an opposing attitude towards father smoking possessed a better smoking-related knowledge. However, male students with disagreement or neutral attitude towards friends smoking were negatively associated with the knowledge of smoking hazards.

**Conclusions:**

The findings of the present study suggest that a significant proportion of male secondary school students in urban Chongqing lack specific knowledge about smoking-related diseases. Targeted educational programs for secondary school students in China are needed to promote tobacco control and tobacco use reduction and cessation among students.

## Background

Smoking causes five million annual deaths globally and 80% of smoking-related deaths occur in low- and middle-income countries (LMICs) every year (World Health Organization 2012). Smoking has been recognized as an independent risk factor for several common chronic diseases such as cardiovascular disease and cancer (Windham et al. 2005; Belchamber et al. 2014). Tobacco use among the youth has become a serious public health problem. According to 2014 China Youth Tobacco Report, about 8000–9000 teenagers become smokers every day in China, about 1.2 million teenagers were exposed to secondhand smoke t, and nearly 7% of middle school students were using tobacco products, and another 19.9% of middle school students tried tobacco products (China CDC 2015). Male smoking prevalence has been high in China. In 2013, 52.9% of adult males and 2.4% of females were tobacco smokers in China (World Health Organization 2013). Furthermore, worldwide it was estimated that males smoked nearly five times as much as females (Guindon and Boisclair 2003), and males smoking rate was higher than females (World Health Organization 2009, 2015; Xu et al. 2015a). Although smoking rate among the adults is decreasing worldwide, the rates are on the rise among male secondary school students. Among middle school students in China, the percent of male students who have tried tobacco products was 30.1% in 2013 (China CDC 2015).

The perception of health risk may not only have a major influence on students’ smoking-related health behavior, but also is an important consideration in health-prevention and programs. In 2014, about 68.4% of students lacked a correct understanding of nicotine addiction (China CDC 2015). In China, approximately 31.6% of students think quitting smoking must be difficult; however, only 18.5% of current smokers among students think that smoking cessation is difficult (China CDC 2015). In the 2010 Global Adult Tobacco China Survey, nonsmokers had a better knowledge about the harms of smoking than smokers (Cheng et al. 2015). Improving knowledge and self-efficacy are essential strategies for reducing the high rate of smoking among teenagers, and previous study found that knowledge of tobacco cessation was significantly positively related with the implementation of tobacco cessation behavioral treatments (Muilenburg et al. 2015). About 13.0% of students believed that smoking makes people look more attractive, and 81% of students had never been taught about smoking or tobacco prevention at school in China (Xu et al. 2015b). Previous studies have also proved that cognition indeed affect behavior (Parasuraman and Jiang 2012; Richardson et al. 2012), and another study among adults found that smoking-related knowledge effectively encouraged smoking cessation, which was showed on the radio, outdoor advertisements or posters, newspapers or magazines/journals, indoor LED screens, mobile TV and television (World Health Organization 2015). Therefore, to prevent smoking and reduce tobacco consumption, it’s more important to pay attention to the smoking-related knowledge and strengthen students’ awareness of smoking-related knowledge (The Ministry of Health of the People’s Republic of China 2012).

A hobby is a regular activity that is done for enjoyment, typically during one’s leisure time. By continually participating in a particular hobby, one can acquire substantial skill and knowledge in that area. A previous study in Japan found the association between smoking behaviors and hobbies (Hu et al. 2007), and another case–control study in Spain found that leisure time activities were associated with the risk of having lung cancer (Ruano-Ravina et al. 2014). In addition, a qualitative analysis found that hobbies were associated with tobacco using and its treatment among young people (Prochaska et al. 2013). Hobbies among students mainly included reading, painting, music; sports, tourism and online games, and different kinds of hobbies might bring different health effects. Computer games have a relationship with teenagers physical health, such as overweight and obesity (Martinovic et al. 2015; Ustinavičienė et al. 2016), but the association with tobacco use still lacks of investigation.

Knowledge, attitudes and practices (KAP) studies have been widely used to investigate health problems in the medical and public health disciplines, which were based on the principle that increasing knowledge, will result in changing attitudes and practices to minimize disease burden (Goh and Chua 2016). Previous studies have found that increased knowledge would help tobacco control, but it still lacks methods to improve smoking-related knowledge. At the same time, the perception of smoking-related health risk among the Chinese has seldom been studied. Currently, more than three quarters of the Chinese did not fully recognize the dangers of smoking (Moore et al. 2014).

The determinants of smoking-related health risk perceptions among secondary school students have not been studied thoroughly in China. Cultural and social context may influence public health risk perceptions (Zhang and Fan 2013). Clarifying the factors that affect smoking-related health risk perceptions is necessary to develop tobacco control strategies among secondary school students in China. In this study we examined smoking-related knowledge, sources of smoking-related knowledge and its influencing factor among male urban secondary school students in a major municipal city in China.

## Methods

### Participants

 The inclusion criteria for participation in the study were (1) student in grades 7–12 in the selected school district; (2) obtaining parental permission and providing assent; (3) male; and (4) being resident of Chongqing. The exclusion criteria were: (1) students with mental illness and (2) writing difficulties. Chinese secondary education included junior middle school and high school. Grades 7–9 comprise junior middle school, and grades 10–12 are high school. Considering the low smoking prevalence in female students in China, this study only focused on male students to investigate typical smoking-related characteristics.

### Ethical approval

We obtained written informed consents from all parents or main caregivers of the enrolled students through parent–teacher conferences. The study was conducted in accordance with the Declaration of Helsinki, and the protocol was approved by the Ethics Committee of Chongqing Medical University.

### Methods

Stratified cluster sampling method was used. Chongqing is one of the municipalities directly under the central government, and it is located in the southwest region of China. After contacting 10 schools in Chongqing, three schools agreed to participate in the study: one school in Bishan County, one in Banan District, and one in Yubei District. Each class from grades 7–12 was randomly selected for data collection. After obtaining the parental informed consents, data were collected through self-administered anonymous questionnaires survey completed by the students.

### Questionnaire

The questionnaire was derived with reference to the China Youth Tobacco Survey (China CDC 2015). We modified the questionnaire according to the results of the pilot study among the target subjects, especially on the presentation of questions and further improved the response options of the questions. Questionnaire domains included demographic characteristics, smoking status, smoking-related knowledge, and parental smoking status.

#### Demographic characteristics

Demographic data included age, school status (middle/high school students), smoking status of parents (non-smoker/smoker), family structure (non-parent families/Single-parent families), per capita household income [high (>5000 ¥), average (1500–5000 ¥), and low (<1500 ¥)] (1 USD = 6.65 ¥).

#### Smoking-related knowledge

Smoking-related knowledge included nine items: (1) whether smoking causes lung cancer (yes/no), whether smoking causes chronic bronchitis (yes/no), whether smoking causes Laryngeal cancer (yes/no), whether smoking causes heart disease (yes/no), whether smoking causes peptic ulcer (yes/no), whether smoking causes cerebral stroke (yes/no); (2) the way that you get smoking-related knowledge [Television (yes/no)/Teachers (yes/no)/Internet (yes/no)/School (yes/no)/Parents (yes/no)/Newspaper (yes/no)/Friends (yes/no)]; (3) nicotine is a kind of addictive substances(yes/no); (4) smoking has a direct effect on the health (yes/no); (5) pregnant women smoking are harmful to fetus (yes/no); (6) smoke-free environment is helpful to health (yes/no); (7) second-hand smoking is harmful to health (yes/no); (8) smoking whether has effects on physiologic, appearance, social and economy(yes/no); (9) your attitude towards family member smoking (Agree/Neutral/Disagree); your attitude towards family smoking (Agree/Neutral/Disagree); and your attitude towards friends smoking (Agree/Neutral/Disagree).

#### Hobbies

To assess hobbies, we asked: “Do you have the following hobbies? Reading (yes/no), music (yes/no), Painting (yes/no), online games (yes/no), sports (yes/no), tourism (yes/no)”.

#### Smoking status

Smoking status was determined by asking respondents whether they were smokers. Current smoker was defined as a person who smoked tobacco at the time of the interview. Assessing the smoking status of parents was conducted the same way. Participants were asked the following questions: “Does your father smoke? (yes/no)” and “Does your mother smoke? (yes/no)”.

### Methods of data collection

In June 2014, a formal survey was conducted in main urban areas of Chongqing. At each survey location, participants who had met the inclusion criteria completed the questionnaires. The investigating team members audited the questionnaire on the spot for completeness and corrected the error before collecting the questionnaires. A total of 1297 completed questionnaires were collected after distributing 1370 questionnaires in the three schools of Bishan Country (300/320), Banan District (526/550) and Yubei District (481/500), with overall response rate of 94.7%.

### Pilot test

The pilot test was conducted with 50 students in a middle school that did not participate in the current study. We modified the questionnaire according to results of the pilot test, especially on the presentation of questions.

### Data collector and training

Nine medical students of Chongqing Medical University were recruited to collect data. The data collectors were trained. Investigation site was coordinated by the investigator. Data collection process entailed explaining to the students that the questionnaire was anonymous and steps were taken to ensure the authenticity and reliability of the questionnaire.

### Data processing and analyses

Frequencies were calculated for all categorical variables and means and standard deviations were computed for all metric variables. Comparison between groups was conducted using χ^2^ test, and generalized estimating equation for statistical analysis. Generalized estimating equation (GEE) (Liang and Zeger 1986; Liu and Zhang 2006) was used to probe factors that affect sources of smoking-related knowledge, which including television, teachers, internet, school, parents, newspaper, and friends. Generalized estimating equation to probe factors affects smoking-related diseases, which including lung cancer, chronic bronchitis, laryngeal cancer, heart disease, peptic ulcer, and cerebral stroke. Statistical tests included a two-sided test, and statistical significance was considered at p < 0.05. All data were analyzed the Statistical Package for Social Sciences (IBM-SPSS), Version 22.0.

## Results

### Characteristics of the sample

Among the participants, 395 (30.5%) were smokers, and the average age of smokers was 16.52 years old. Smoking rates of high school students were higher than middle school students (p < 0.05). Smokers’ father’s smoking rates were statistically significantly higher than non-smokers’ (smoker vs. non-smoker = 6.3 vs. 3.1%, p < 0.05). Smokers’ mother’s smoking rates were higher than non-smokers’ (smoker vs. non-smoker = 50.2 vs. 64.6%, p < 0.05). Statistically significant differences were observed on “per capita household income (p = 0.003)”, “the number of smokers in family (p < 0.0001)”, “the number of smokers among friends (p < 0.001)” between smokers and non-smokers. Non-smokers showed more unfavorable attitude towards both family members and friends smoking than smokers (p < 0.001). Among smokers, only 25.8% students disagreed with friends smoking and 50.1% students disagreed with family member smoking, with more concern for family members’ health (see Table 1).

**Table 1:** Characteristics of the study participants stratified by smoking status in Chongqing, China [%/mean (SD)]

Variable	Smokers (n = 395)	Non-smokers (n = 902)	Statistical test
Age (mean ± SD)	16.52 ± 1.40	15.35 ± 1.89	t = 1.83, p < 0.001*
School category			χ^2^ = 42.4823, p < 0.001*
Middle school	61 (15.4)	298 (33.0)	
High school	334 (84.6)	604 (67.0)	
Marital status of parents			χ^2^ = 2.011, p < 0.366
Non-single parent families	323 (81.8)	760 (84.3)	
Single parent families	69 (17.5)	139 (15.4)	
Missing	3 (0.8)	3 (0.3)	
Family economic situation			χ^2^ = 11.5954, p = 0.003*
Rich	57 (14.4)	171 (19.0)	
Average	244 (61.8)	583 (64.6)	
Impoverished	94 (23.8)	148 (16.4)	
Father smoking status			χ^2^ = 22.7722, p < 0.001*
Non-smoker	140 (35.4)	449 (49.8)	
Smoker	255 (64.6)	453 (50.2)	
Mother smoking status			χ^2^ = 7.2892, p = 0.007*
Non-smoker	370 (93.7)	874 (96.9)	
Smoker	25 (6.3)	28 (3.1)	
Number of family member smoking			χ^2^ = 33.0289, p < 0.001*
None	80 (20.3)	310 (34.4)	
One to two	257 (65.1)	520 (57.7)	
Three and above	58 (14.7)	72 (8.0)	
Number of friends smoking			χ^2^ = 236.0684, p < 0.001*
None	82 (20.8)	582 (64.5)	
One to two	48 (12.2)	99 (11.0)	
Three and above	265 (67.1)	221 (24.5)	
Attitude towards friends smoking			χ^2^ = 155.8387, p < 0.001*
Agree	47 (11.9)	24 (2.7)	
Neutral	246 (62.3)	323 (35.8)	
Disagree	102 (25.8)	555 (61.5)	
Attitude towards family member smoking			χ^2^ = 70.4818, p < 0.001*
Agree	29 (7.3)	20 (2.2)	
Neutral	168 (42.5)	222 (24.6)	
Disagree	198 (50.1)	660 (73.2)	

* Statistically significant (p < 0.05)

### Hobbies and smoking

Non-smokers’ hobbies of reading were significantly higher than smokers’ (χ^2^ = 11.5845, p = 0.0007). Smokers’ hobbies of online games (χ^2^ = 14.9106, p = 0.0001), and sports (χ^2^ = 4.8609, p = 0.0275) were significantly higher than non-smokers’ (see Table 2).

**Table 2:** Hobbies stratified by smoking status in Chongqing, China (n, %)

Hobbies	Smokers (n = 395)	Non-smokers (n = 902)	Statistical test
Reading	154 (39.0)	444 (49.2)	χ^2^ = 11.5845, p = 0.001*
Music	268 (67.9)	588 (65.2)	χ^2^ = 0.8659, p = 0.352
Painting	83 (21.0)	209 (23.2)	χ^2^ = 0.7334, p = 0.392
Online games	268 (67.9)	509 (56.4)	χ^2^ = 14.9106, p < 0.001*
Sports	262 (66.3)	540 (59.9)	χ^2^ = 4.8609, p = 0.028*
Tourism	180 (45.6)	424 (47.0)	χ^2^ = 0.228, p = 0.633

* Statistically significant (p < 0.05)

### Smoking-related knowledge

Less than twenty percent of students knew that smoking can cause Heart disease (Smokers vs. Non-smokers = 19.5 vs. 19.7%), Peptic ulcer (Smokers vs. Non-smokers = 16.7 vs. 12.5%), and cerebral stroke (Smokers vs. Non-smokers = 11.1 vs. 12.2%). The perception among smokers of “Smoke-free environment is helpful to health (p = 0.013)”, “Second-hand smoke is harmful to health (p = 0.007)”, “Smoking on physiology, appearance, short-term and long-term impact on society and economy (p = 0.002)”, “Personal, family, peers and the media can affect people decide whether or not to smoke (p = 0.041)” is statistically significantly lower than the non-smokers (see Table 3).

**Table 3:** Smoking-related disease stratified by smoking status in Chongqing, China (n, %)

Variable	Smokers (n = 395)	Non-smokers (n = 902)	Statistical test
Smoking-related diseases	–	–	–
Lung cancer	383 (97.0)	877 (97.2)	χ^2^ = 0.070, p = 0.790
Chronic bronchitis	227 (57.5)	499 (55.3)	χ^2^ = 0.5138, p = 0.474
Laryngeal cancer	223 (56.5)	457 (50.7)	χ^2^ = 3.693, p = 0.055
Heart disease	77 (19.5)	178 (19.7)	χ^2^ = 0.01, p = 0.920
Peptic ulcer	66 (16.7)	113 (12.5)	χ^2^ = 4.036, p = 0.045*
Cerebral stroke	44 (11.1)	110 (12.2)	χ^2^ = 0.2927, p = 0.589
Smoking-related perceptions	–	–	–
Nicotine is a kind of addictive substances	316 (80.0)	744 (82.5)	χ^2^ = 1.1344, p = 0.287
Smoking has a direct effect on the health	348 (88.1)	812 (90.0)	χ^2^ = 1.0729, p = 0.300
Pregnant women smoking is harmful to fetus	371 (93.9)	861 (95.5)	χ^2^ = 1.3517, p = 0.245
Smoke-free environment is helpful to health	356 (90.1)	848 (94.0)	χ^2^ = 6.2345, p = 0.013*
Second-hand smoking is harmful to health	357 (90.4)	852 (94.5)	χ^2^ = 7.2197, p = 0.007*
Smoking has effects on physiologic, appearance, social and economy	276 (69.9)	702 (77.8)	χ^2^ = 9.3701, p = 0.002*
Whether a person smoke depends on the feeling of individual, family, peers and the media	274 (69.4)	675 (74.8)	χ^2^ = 4.1815, p = 0.041*

* Statistically significant (p < 0.05)

### Sources of smoking-related knowledge

Sources of smoking-related knowledge among smokers were 302 (76.46%) TV, 277 (70.13%) teachers and 263 (66.58%) network. Sources of smoking-related knowledge among non-smokers were 692 (76.72%) TV, 659 (73.06%) network and 574 (63.64%) school, 563 (62.42%) teachers, 556 (61.64%) newspaper, 534 (59.2%) parents and 306 (33.92%) friends. Smokers’ smoking-related knowledge was obtained from the teacher, were significantly higher than non-smokers (p < 0.05). Non-smokers were statistically significantly higher on “smoking-related knowledge was obtained from the network” than smokers (p < 0.05) (see Table 4).

**Table 4:** Sources of getting smoking-related knowledge stratified by smoking status in Chongqing, China (n, %)

The way you get smoking-related knowledge	Smokers (n = 395)		Non-smokers (n = 902)		Statistical tests
	Yes	Rank	Yes	Rank	
Television	302 (76.5)	1	692 (76.7)	1	χ^2^ = 0.0106, p = 0.918
Teachers	277 (70.1)	2	563 (62.4)	4	χ^2^ = 7.1553, p = 0.008*
Internet	263 (66.6)	3	659 (73.1)	2	χ^2^ = 5.608, p = 0.018*
School	250 (63.3)	4	574 (63.6)	3	χ^2^ = 0.0141, p = 0.905
Parents	225 (57.0)	5	534 (59.2)	6	χ^2^ = 0.5677, p = 0.451
Newspaper	219 (55.4)	6	556 (61.6)	5	χ^2^ = 4.3877, p = 0.918
Friends	149 (37.7)	7	306 (33.9)	7	χ^2^ = 1.7389, p = 0.187

* Statistically significant (p < 0.05)

### Generalized estimating equation to probe factors that affect sources of smoking-related knowledge

Generalized estimating equations found that students whose father smoked has less way to get tobacco-related knowledge (odds ratio, OR = e^−0.202^ = 0.82, p < 0.05). Students whose mother smoked has less way to get tobacco-related knowledge (OR = e^−0.354^ = 0.70, p < 0.05) (see Table 5).

**Table 5:** Generalized estimating equations parameter estimates for the way to get smoking-related knowledge in Chongqing, China

Parameter	Category	B	SE	95% CI	p value
Intercept	–	0.15	0.136	−0.117 to 0.416	0.270
School category	High school	0.05	0.053	−0.054 to 0.154	0.346
	Middle school	0^a^			
Family financial circumstances	Rich	−0.005	0.074	−0.151 to 0.14	0.944
	Average	−0.096	0.059	−0.211 to 0.019	0.102
	Impoverished	0^a^			
Father’s smoking status	Smoker	−0.202	0.061	−0.321 to −0.083	0.001*
	Non-smoker	0^a^			
Mother’s smoking status	Smoker	−0.354	0.114	−0.578 to −0.131	0.002*
	Non-smoker	0^a^			
The number of smokers in family	Three or more people	0.12	0.093	−0.063 to 0.302	0.199
	One or two people	0.103	0.068	−0.029 to 0.236	0.127
	None	0^a^			
The number of smokers among friends	Three or more people smoke	0.051	0.052	−0.05 to 0.153	0.324
	One or two people	0.094	0.074	−0.05 to 0.239	0.201
	None	0^a^			
You altitude towards smoking friends	Disagree	0.235	0.132	−0.023 to 0.493	0.075
	Neutral	0.213	0.128	−0.038 to 0.464	0.096
	Agree	0^a^			
You altitude towards smoking family	Disagree	0.181	0.153	−0.12 to 0.481	0.238
	Neutral	0.097	0.153	−0.203 to 0.397	0.527
	Agree	0^a^			

* Statistically significant (p < 0.05)

^a^Set to zero because this parameter is redundant

### Generalized estimating equation to probe factors affect knowledge of smoking-related diseases

Generalized estimating equations found that male students who were high school were 1.13 times more to acquire knowledge of smoking-related diseases (OR = e^0.123^ = 1.13, p < 0.05). Students who come from economically rich family condition (OR = 1.25) and disagree with family member smoking (OR = 1.80) were more likely to acquire higher knowledge of smoking-related diseases. Students who disagreed or were neutral towards friends smoking were less likely to acquire higher knowledge of smoking-related disease (OR = 0.69) (see Table 6).

**Table 6:** Generalized estimating equation to probe factors affect knowledge of smoking-related diseases in Chongqing, China

Parameter	Category	B	SE	95% CI	p value
Intercept		−0.679	0.151	−0.974 to −0.384	0
School category	High school	0.123	0.057	0.011–0.235	0.032*
	Middle school	0^a^			
Family financial circumstances	Rich	0.222	0.079	0.067 to 0.376	0.005*
	Average	0.038	0.063	−0.086 to 0.162	0.547
	Impoverished	0^a^			
Father’s smoking status	Smoker	−0.095	0.064	−0.221 to 0.031	0.139
	Non-smoker	0^a^			
Mother’s smoking status	Smoker	0.001	0.127	−0.248 to 0.25	0.995
	Non-smoker	0^a^			
The number of smokers in family	Three or more persons	−0.082	0.099	−0.277 to 0.113	0.411
	One or two persons	0.025	0.071	−0.115 to 0.165	0.723
	None	0^a^			
The number of smokers among friends	Three or more	0.044	0.055	−0.065 to 0.152	0.43
	One or two	0.069	0.077	−0.082 to 0.221	0.37
	None	0^a^			
You attitude towards to friends smoke	Disagree	−0.371	0.142	−0.649 to −0.092	0.009*
	Neutral	−0.278	0.138	−0.549 to −0.007	0.044*
	Agree	0^a^			
You attitude towards family members smoke	Disagree	0.588	0.168	0.259–0.917	0.000*
	Neutral	0.445	0.168	0.116–0.774	0.008*
	Agree	0^a^			

* Statistically significant (p < 0.05)

^a^Set to zero because this parameter is redundant

## Discussion

The findings of this study show that many middle school students have cognitive limitations on the specific knowledge of smoking-related diseases. About 95% of high school students knew that smoking was harmful to health, which was a little higher than the awareness rate in Bangalore, India, where 90.2% students knew the harmful effects of tobacco in 2015 (Singh et al. 2015). Although more than 90% students knew smoking can cause lung cancer, <20% know other diseases (heart disease, peptic ulcer, cerebral stroke) caused by smoking. Students in the specific understanding of the dangers of smoking are not enough, especially in smokers. Most people know that smoking is harmful to health but they did not know what it exactly harms. Therefore, it is necessary to promote the knowledge of smoking in many ways, especially in smokers.

Main sources to receive tobacco-related knowledge are TV and the Internet. However, still 23.36% of the students indicate that they did not get smoking-related knowledge from these sources. It is still very necessary for the improvement of the coverage of smoking-related knowledge. This study further confirms that smoking-related knowledge was more likely to be noticed on TV than on other media among the urban secondary school students (World Health Organization 2015). This suggests that tobacco control has a certain effect, but there is stillroom for improvement of the coverage. Tobacco-control mass media campaigns could encourage people to refrain from smoking (Farrelly et al. 2007). This survey showed that <40% of high school students get smoking-related knowledge from friends. In this case, when nonsmoking youth are friends with smokers and have weak relationships with family and school faculty, they are more likely to become smokers themselves than are youth who do not associate with smokers (Jordan et al. 2014). This study found that students whose father smoke and mother smoke received less tobacco-related knowledge. Previous research has shown that father smoking or mother smoking is a factor affecting teenagers smoking (Jordan et al. 2014); besides, with father or mother smoked, teenagers get less knowledge about tobacco which might be one of the causes of tobacco initiation. To improve the coverage of tobacco control, governments should make full use of the TV, Internet and other media for tobacco control propaganda. In addition, the way to improve the awareness rate of the hazards of smoking ought to be taken into consideration.

This study found that students were from high school, with rich family economic condition and with an opposing attitude toward smoking have a better understanding of the smoking-related knowledge. This survey also showed that 63% of students got smoking-related knowledge from school. Previous studies show that school intervention activities about tobacco control played a positive role in improving middle school students’ awareness of knowledge about tobacco control; the right beliefs hold rate and health behavior formation rate of tobacco (Zawahir et al. 2013). Previous study also showed that 81% of students had never been taught throughout school for smoking or tobacco prevention (Muilenburg et al. 2015). Considering this, the schools should systematically offer tobacco control education within the regular curriculum. Community programs, school and college policies and interventions that encourage tobacco-free environments and lifestyles can reduce smoking rates (U.S. Department of Health and Human Services 2012). Compared with students from low socioeconomic status background, male students with rich family economic background would receive more knowledge about the harm of tobacco using from parents (Bird et al. 2016), and those supported measures would prevent their smoking initiation. Previous study found that parents who believed that quitting would benefit their child were more likely to quit smoking (Mahabee-Gittens et al. 2014). Similarly, out of the strongly concern for family members health, male students who disagreed with family members smoking were associated with more knowledge of smoking hazards. A systematic review and meta-analysis found that family-based interventions prevent children and adolescents from starting to smoke (Thomas et al. 2016a). Also, good family relationships may help to reduce tobacco use among parents and children. However, the situation is different in the attitude towards friends smoking. Students who approved friends smoking possessed a better knowledge about the harm of smoking. The potential interpretation is that only a few students disagreed with friends smoking and the amount of smokers remained neutral with friends smoking, with less concern for friends smoking status and friends physical health. Besides, smoking initiation was positively associated with having more friends that smoke (Huisman 2014), and previous finding suggested that friends tended to select each other on similar smoking behavior (Voorhees et al. 2011), which might explain that the amount of smokers knowing the hazards of smoking still hold the neutral attitude towards friends smoking and may even they agreed with friends smoking.

Hobbies may affect secondary school students smoking. The study found that non-smokers’ hobbies of reading were statistically significantly higher than smokers’, and smokers’ hobbies of online games and sports were statistically significantly higher than non-smokers’. A previous study in European found that smokers visited more unsupervised places where smoking was permitted, whereas nonsmokers visited libraries or other more supervised places (De Vries et al. 2007). Especially in computer games rooms or playground, male students lack the adult supervision and guidance which are necessary to ensure that youth do not smoke. Besides, parents who know about the adolescent’s free time activities in the past 30 days has a protective effect on the adolescent (Thomas et al. 2016b), because family supervision is important to prevent male students from using tobacco. These findings indicate that tobacco control among secondary students should pay more attention to the students who have hobbies of online games and sports.

This study has several limitations. Firstly, the instrument was primarily measuring attitudes and was based on self-reports. It would not have been possible to use any other means to measure them. Secondly, 72.3% of subjects were high school students, only 27.7% of subjects were middle school students and this outcome may affect the representativeness of secondary school students, which requires cautious interpretations of the study results, especially among middle school students. Thirdly, our study only investigates secondary students in the city, and is not representative of rural secondary school students. In China, smoking prevalence was higher among rural secondary students (China CDC 2015). It would be useful to replicate our work in rural secondary school students. Fourthly, the design of this study was cross-sectional and as such nothing can be said about temporality of the association. The predictive direction of proposed relationships can be ascertained by longitudinal studies.

## Conclusions

The findings of the current study suggest that many Chinese secondary school students have cognitive limitations on the specific knowledge of smoking-related diseases. Students whose father smoke and mother smoke have received less tobacco-related knowledge. High school students with rich family economic condition and with a positive attitude towards smoking had better understanding of the smoking-related knowledge. Male students who disagree or were neutral towards friends smoking were in a negative correlation with the knowledge of smoking hazards. The findings of the present study will contribute to a better understanding of the smoking-related knowledge among secondary school students and will allow for the development of health-prevention programs for urban male secondary school students in China. Targeted educational programs for secondary school students in China are needed to promote tobacco control and tobacco use reduction and cessation among students.
